# Supplementary material for: Stable clonal contribution of lineage-restricted stem cells to human hematopoiesis
Source: Nat Genet. 2025 Nov 11;57(12):3088–100. doi: 10.1038/s41588-025-02405-w (PMC12695654; doi:10.1038/s41588-025-02405-w)
Supplement: Supplementary file 1 — Supplementary Figs. 1–5 [file 41588_2025_2405_MOESM1_ESM.pdf]

nature genetics

Supplementary information https://doi.org/10.1038/s41588-025-02405-w

Stable clonal contribution of lineage-restricted stem cells to human hematopoiesis

In the format provided by the authors and unedited

Supplementary Notes

Supplementary Methods

Error-corrected targeted DNA capture sequencing (ECTS) analysis

Parameters for mutation calling by EBCall (https://github.com/friend1ws/EBCall):

(i) Mapping quality threshold = 30

(ii) Base quality threshold = 15

(iii) Minimum depth in BM = 8

(iv) Minimum number of variant reads in BM = 3

(v) Minimum variant allele frequency in sample = 0.00005

(vi) EBCall P-value threshold = 10^{-2}

(vii) samtools mpileup option of UNMAP,SECONDARY,QCFAIL

After mutation calling, the following candidates were removed:

(i) Outside of target regions ±10 bp

(ii) Strand ratio = 1 or 0

(iii) Number of alternative reads in BM <10

(iv) EBCall P-value ≥10^{-5}

(v) Variant allele frequency (VAF) in bone marrow (BM) ≤99% confidence interval (CI) of VAF in normal control samples, based on 10,000 bootstrap replicates

(vi) VAF by base counts / VAF by reads count ≥0.5 and ≤2

Finally, candidates fulfilling the either one of the following criteria were deemed to be somatic mutations:

(i) Reported in ≥10 hematological malignancy cases in COSMIC v91

(ii) Frequency <0.1% in all four SNP databases (1000g2015aug, esp6500siv2, HGVD_20160412, ExAC), and fulfilling one of the following criteria:

a. BM VAF <0.3 or VAF 0.7–0.9
b. Nonsense, frameshift or splicing sites mutations
c. Pathogenic in ClinVar database

Whole-exome sequencing

Parameters for mutation calling and filtering:

(i) Mapping Quality score ≥20
(ii) Base Quality score ≥15
(iii) Number of total reads ≥8
(iv) Number of variant reads ≥5
(v) VAF in paired buccal swab <0.2
(vi) VAF in other non-paired normal controls <0.05 in all
(vii) Mean VAF in other non-paired normal controls <0.01
(viii) Strand ratio in tumor ≠ 0 or 1
(ix) VAF by base counts / VAF by reads count ≥0.5 and ≤2
(x) Indel_mismatch_count ≤5 & indel_mismatch_rate<0.05
(xi) P-value by Fisher <0.1
(xii) P-value by EBFilter <0.001
(xiii) Within targeted regions ±50 bp
(xiv) Coverage ≤1000
(xv) Not located within repeat regions

Single cell derived colony whole-genome sequencing (WGS) analysis

Parameters for mutation calling and filtering:

(i) Mapping Quality score ≥20
(ii) Base Quality score ≥15

(iii) Number of total reads ≥8

(iv) Number of variant reads ≥4

(v) VAF in colonies ≥0.25

(vi) VAF in paired buccal swab <0.1

(vii) VAF in all non-paired normal controls <0.1

(viii) Mean VAF in other non-paired normal controls <0.02

(ix) Strand ratio in tumor ≠ 0 or 1

(x) VAF by base counts / VAF by reads count ≥0.5 and ≤2

(xi) P-value by Fisher <0.1

(xii) P-value by EBFilter[54] <0.001

(xiii) Locates on nuclear DNA

(xiv) No other candidates exist within a range of ≥2 and ≤50 bases

(xv) Frequency is ≤0.01% in all the four SNP database (1000g2015aug, esp6500siv2, HGVD_20160412, ExAC) and fall in at least one of the following:

   a. Candidates in non-repetitive regions
   b. Nonsense, frameshift or splicing sites mutations
   c. Pathogenic in ClinVar database
   d. Deleterious in SIFT_pred, probably damaging or possibly damaging in Polyphen2_HVAR_pred, or high or medium in MutationAssessor_pred

To eliminate mutations potentially acquired as a result of cell culture, only mutations with VAFs >25% were used for the analysis.

Structural variants were identified through GenomonSV (https://github.com/Genomon-Project/GenomonSV) with the following setting (--min_junc_num 2 --max_control_variant_read_pair 10 --min_overhang_size 50 --thread_num 6) with paired mode using buccal swab DNA. After calling, the following filters were applied.

(i) Allele frequency in colony >0.2

(ii) Allele frequency in paired colony = 0

(iii) Number of variant reads in colony ≥5

(iv) Number of variant reads in paired normal control ≤ 1

(v) Depth in colony ≥10

(vi) Depth in normal control ≥10

(vii) Size for the inversion >1000

(viii) Size for the overhang of supporting read on the breakpoint: >100

(ix) P-value by Fisher <0.01

(x) Not observed in non-paired controls

Since break points of structural variations can be ambiguous, structural variants with break points of ±5 bp from the same donors were treated as the same structural variants. If a structural variant excluded in one colony but passing filtering criteria with an allele frequency of >0.1 in another colony derived from the same case, the structural variant was rescued as it was deemed likely to be present also in the colony not passing the original filtering criteria.

Calculation of SNP sensitivity in single colony whole-genome sequencing data

Because sequence depth affects the number of mutations in each colony, we evaluated the sensitivity of single nucleotide polymorphisms (SNPs) detection in each colony by calling SNPs only on exonic regions with the same setting as above but without paired normal controls.

(i) Number of alternative reads ≥4

(ii) VAF in colony ≥0.25 & VAF in non-paired normal controls <0.05

(iii) P-value by EB Call <0.001

(iv) VAF by base count / VAF by read count ≥0.5 & ≤2

(v) Candidates on non-repeated regions

(vi) Registered in 1000g2015aug SNP database

The binary SNP matrix was then created and sensitivity calculated with the following criteria (VAF ≥0.15 & mutant reads≥2 to positive, mutant reads=0 & ≥5 total reads to negative, and the rest to unknown).

Supplementary Fig. 1

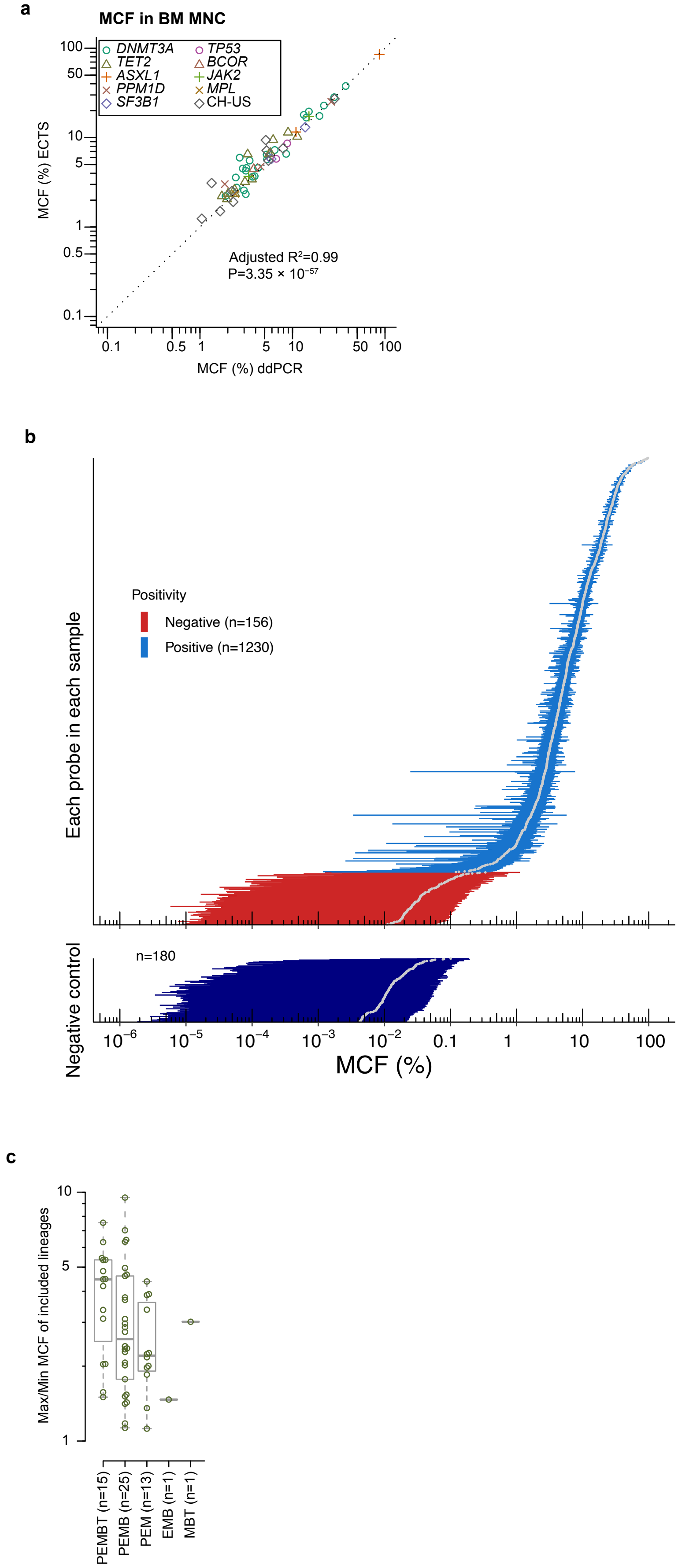

Supplementary Fig. 1. Validation of ddPCR results

a. Comparison of MCF in BM MNCs obtained between droplet digital PCR (ddPCR) and error-corrected targeted DNA capture sequencing (ECTS) for all mutations with ≥2% and ≥1% MCF for CH-driver (n=51) and CH-US mutations (n=10), respectively, subjected to ddPCR analysis. R square value adjusted for the number of predictors and P-value of linear model are provided.

b. A line plot showing the mean (grey dots) and 95% credible intervales of each sorted HSC and blood lineage sample estimated using Bayesian inference, accounting for the number of ddPCR events and the number of sorted cells across all samples used in the manuscript (See Methods) (top). Each bar represent data from an individual sample for each ddPCR probe (blue: judged as positive, n=1217, red: judged as negative, n=157). Samples with fewer number of sorted cells exhibit wider credible intervals, particularly for MkP (**Extended Data Fig. 5**). A line plot for negative controls (peripheral blood DNA from anonymized donors, n=180) (bottom).

c. Fold changes of maximum MCF against minimum MCF among biased lineages for each clone for the different HSC lineage contribution patterns. The center lines and boxes denote median values and the first and third quartiles, respectively. Whiskers indicate maximum and minimum values within 1.5 interquartile range.

Supplementary Fig. 2

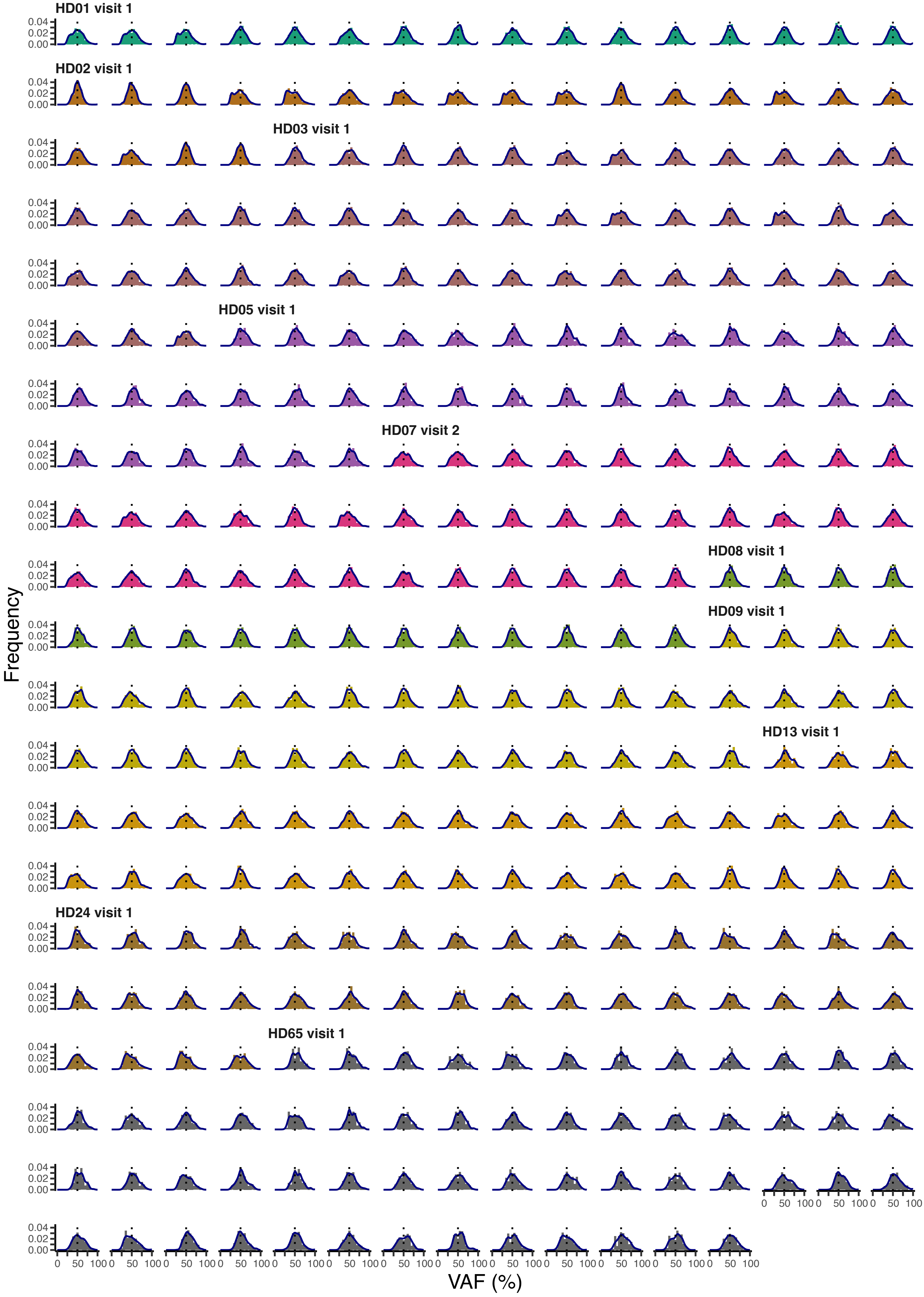

Supplementary Fig. 2. Variant allele frequency (VAF) distribution of mutations detected through single colony whole-genome sequencing

Histogram showing VAF distribution of detected mutations through WGS of single-HSPC derived colonies subjected to the phylogenetic analysis (n=333) from 10 donors as indicated by different colors. Vertical dotted lines are plotted on 50%. Normal distribution of VAF around 50% in all colonies confirms single cell origin and no significant skewing during PCR amplification through library preparation.

Supplementary Fig. 3

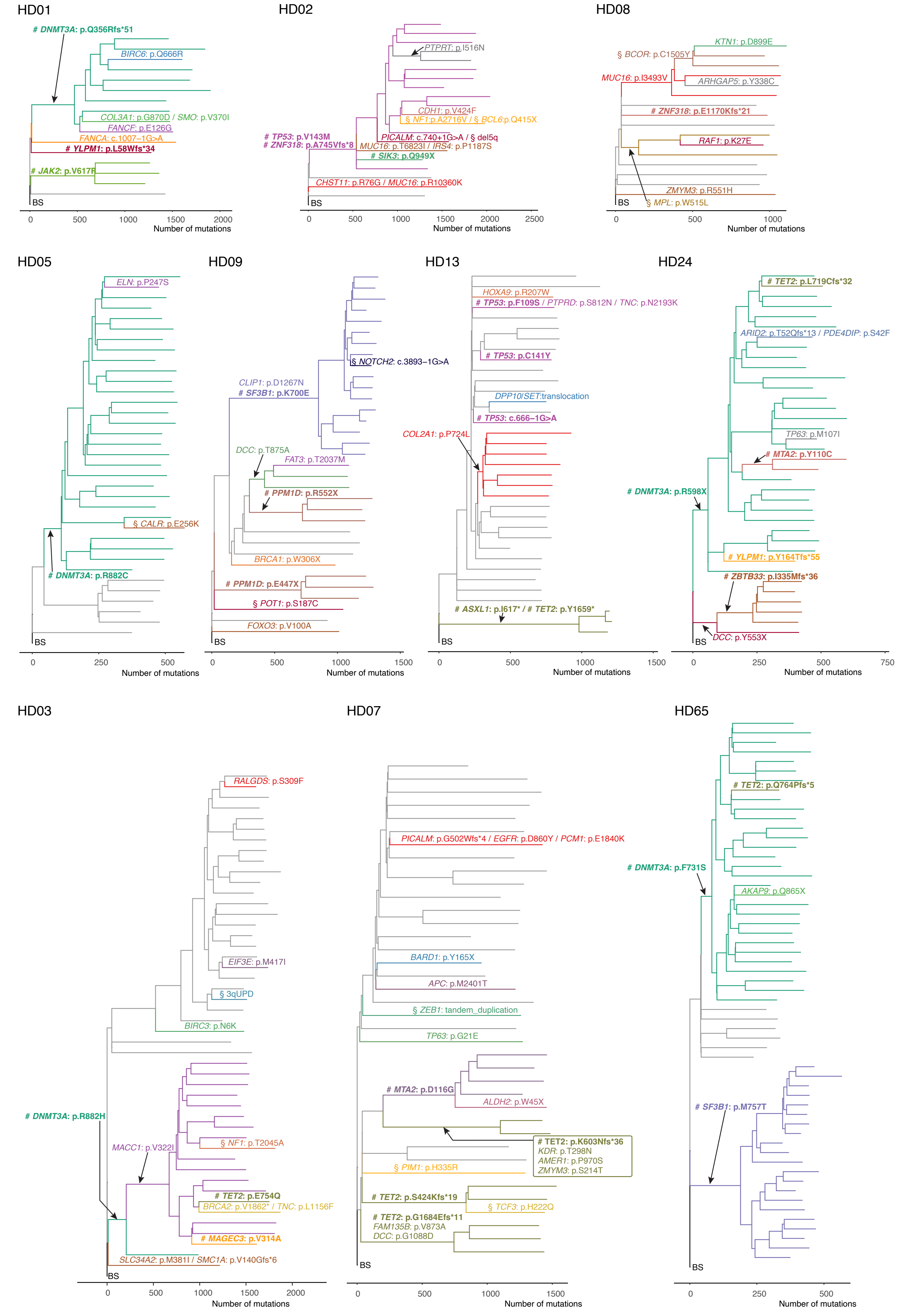

Supplementary Fig. 3. Phylogenetic history of candidate cancer driver mutations

Phylogenetic trees from 10 healthy donors (HDs 01, 02, 03, 05, 07, 08, 09, 13, 24, and 65) constructed from WGS results in the scale of the number of mutations corrected by sequencing depth in each donor (See **Methods**). Presence and positions in phylogenetic trees of CH-driver mutations and possible myeloid or cancer driver mutations, structural variations, or chromosomal abnormalities are shown. DNA from buccal swab (BS) was used as germline controls. The WGS analysis confirmed that most of the expanded clones are driven by common CH-driver mutations—indicated by a '#' before the mutated gene names and their corresponding amino acid changes, which are shown in bold in the figure—, by other myeloid driver mutations indicated by a '§', or by no identifiable driver candidates, rather than by other candidate cancer driver mutations. The only exceptions include a *COL2A1* p.P724L positive clone in HD13 and a *MUC16* p.I3493V positive clone in HD08; the latter harbored a myeloid driver *BCOR* p.C1505Y mutated clone as a subclone.

Supplementary Fig. 4

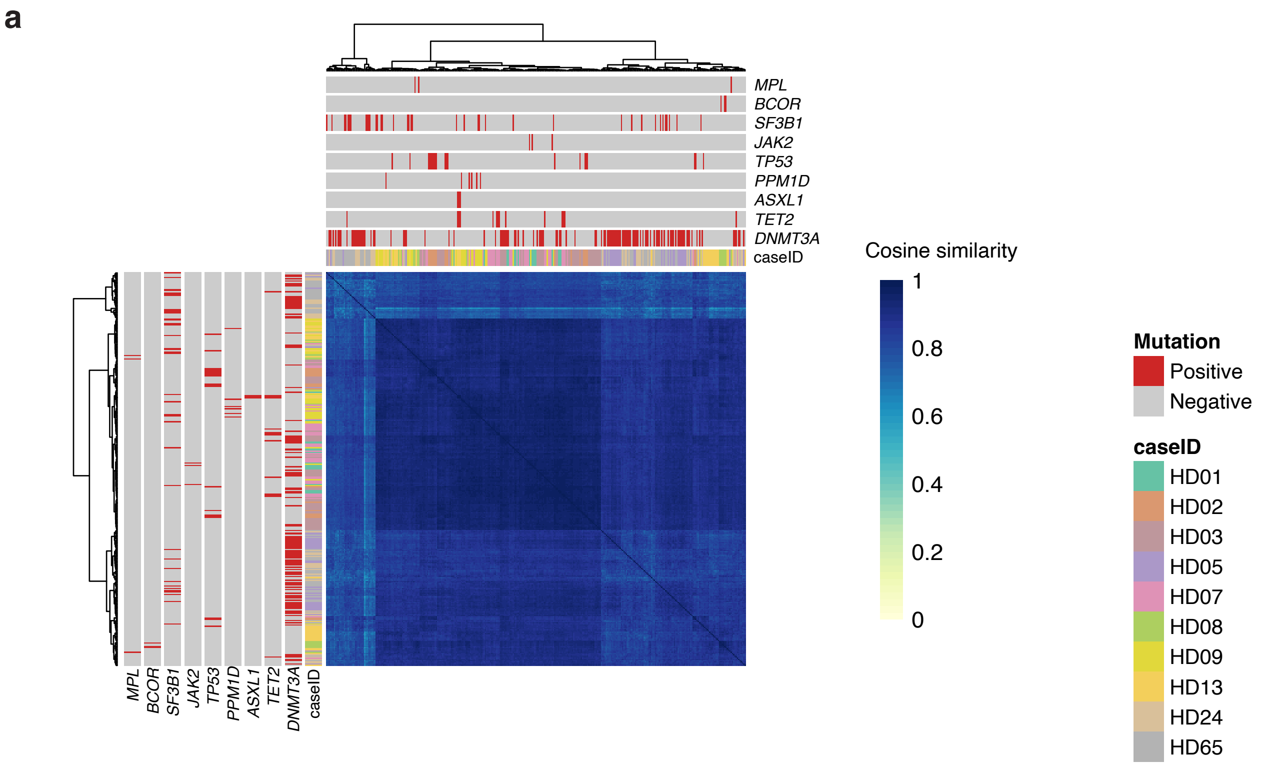

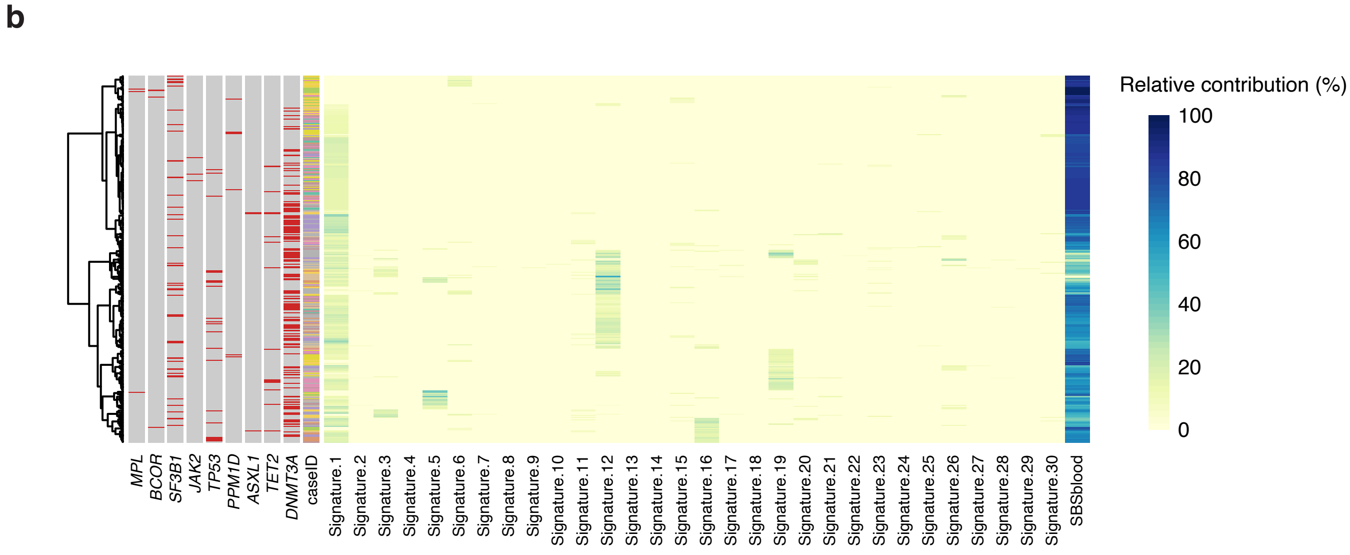

Supplementary Fig. 4. Impact of driver mutations on mutational signatures

a. Cosine similarities of 96 patterns in trinucleotides between all sequenced colonies derived from single HSCPs from 10 healthy donors for single base substitutions (SBS). Driver mutation status (positive or negative) and donor ID in each colony are indicated.

b. Relative contribution of mutations allocated to the 30 COSMIC v2 signature and SBS-blood patterns in each colony.

Supplementary Fig. 5

a

Age at the midpoint of the branch for CH-US mutations

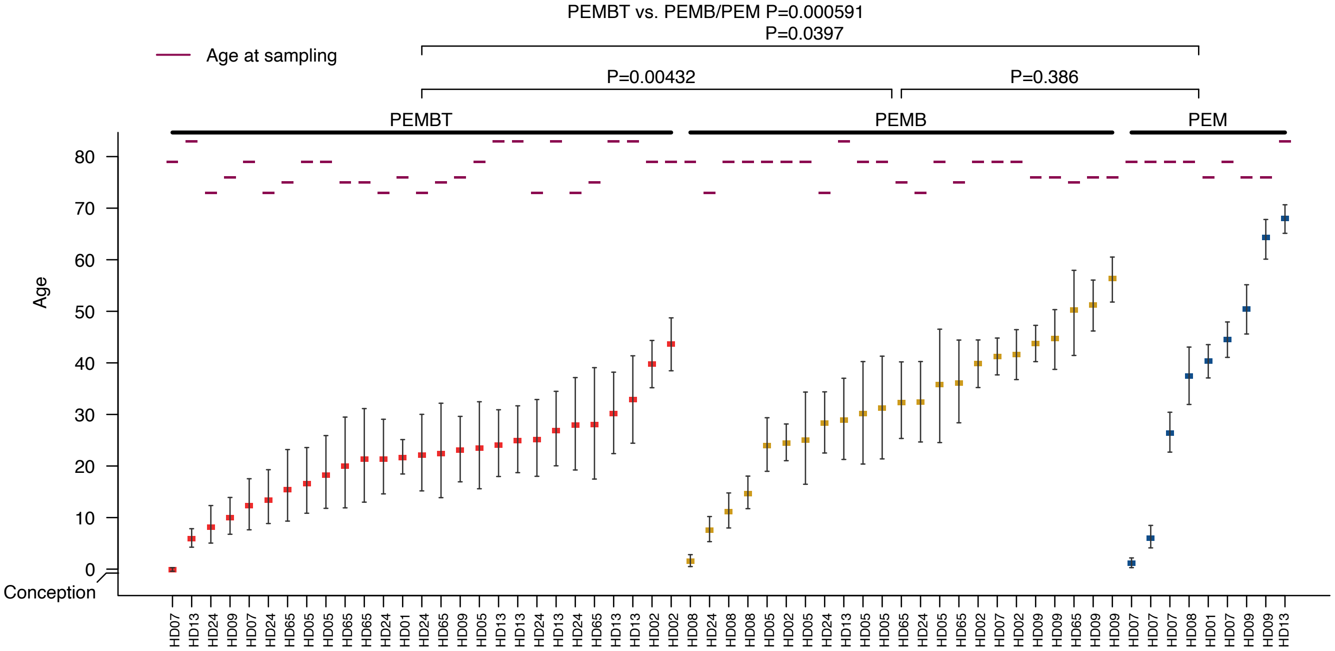

b

Age at the proximal end of the branch for CH-US mutations

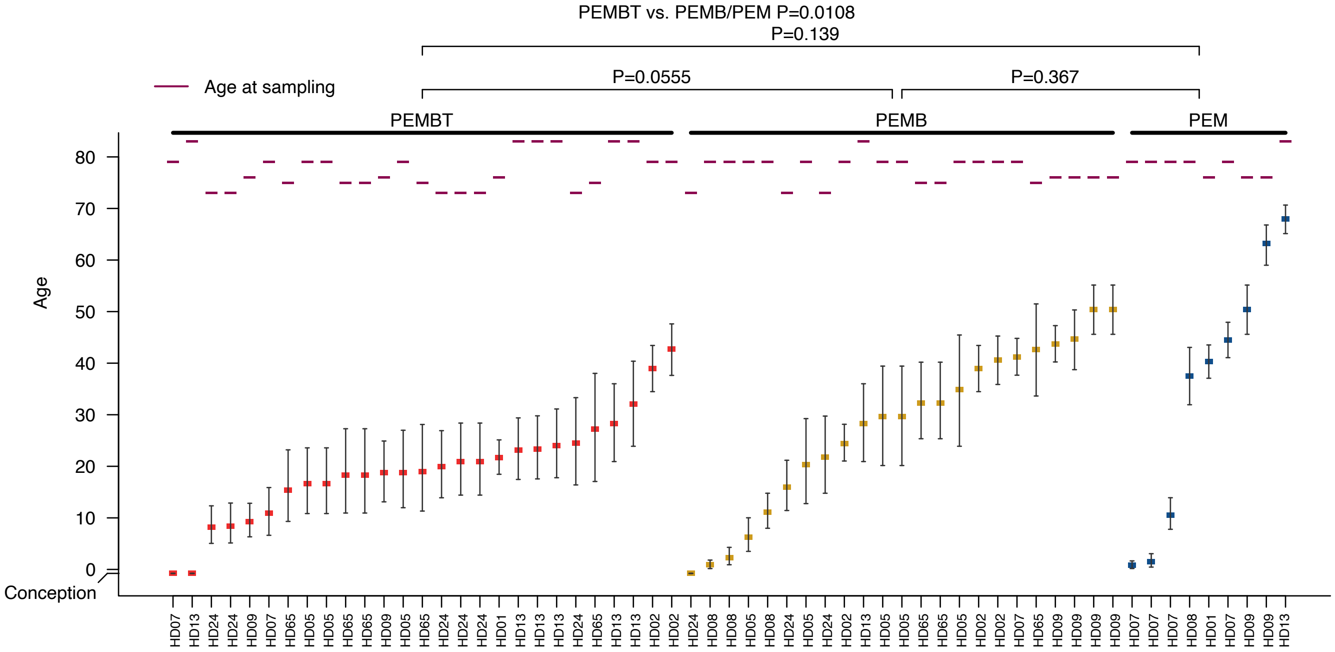

Supplementary Fig. 5. Evaluation of estimation of acquisition dating for HSC clonal mutations

The same analysis as in **Fig. 3c** was performed, except rather assuming that acquisition of CH-US HSC mutations occurred at the midpoint (**a**) or the proximal end (**b**) of the branch, instead of the distal end (see **Methods**).
