# Supplementary material for: Stable clonal contribution of lineage-restricted stem cells to human hematopoiesis
Source: Nat Genet. 2025 Nov 11;57(12):3088–100. doi: 10.1038/s41588-025-02405-w (PMC12695654; doi:10.1038/s41588-025-02405-w)
Supplement: Supplementary file 2 — Reporting Summary [file 41588_2025_2405_MOESM2_ESM.pdf]

Corresponding author(s): Tetsuichi Yoshizato
Sten Eirik W. Jacobsen

Last updated by author(s): Oct 4, 2025

Reporting Summary

Nature Portfolio wishes to improve the reproducibility of the work that we publish. This form provides structure for consistency and transparency in reporting. For further information on Nature Portfolio policies, see our Editorial Policies and the Editorial Policy Checklist.

Statistics

For all statistical analyses, confirm that the following items are present in the figure legend, table legend, main text, or Methods section.

n/a	Confirmed	
☐	☒	The exact sample size (n) for each experimental group/condition, given as a discrete number and unit of measurement
☐	☒	A statement on whether measurements were taken from distinct samples or whether the same sample was measured repeatedly
☐	☒	The statistical test(s) used AND whether they are one- or two-sided<br>*Only common tests should be described solely by name; describe more complex techniques in the Methods section.*
☐	☒	A description of all covariates tested
☐	☒	A description of any assumptions or corrections, such as tests of normality and adjustment for multiple comparisons
☐	☒	A full description of the statistical parameters including central tendency (e.g. means) or other basic estimates (e.g. regression coefficient) AND variation (e.g. standard deviation) or associated estimates of uncertainty (e.g. confidence intervals)
☐	☒	For null hypothesis testing, the test statistic (e.g. F, t, r) with confidence intervals, effect sizes, degrees of freedom and P value noted<br>*Give P values as exact values whenever suitable.*
☐	☒	For Bayesian analysis, information on the choice of priors and Markov chain Monte Carlo settings
☒	☐	For hierarchical and complex designs, identification of the appropriate level for tests and full reporting of outcomes
☐	☒	Estimates of effect sizes (e.g. Cohen's d, Pearson's r), indicating how they were calculated

Our web collection on statistics for biologists contains articles on many of the points above.

Software and code

Policy information about availability of computer code

Data collection	Flow cytometry data: FACSDiva (BD version 8.0.2)<br>DNA sequencing data: NovaSeq 6000 (Illumina)<br>ddPCR data: QuantaSoft version 1.7.4 and QuantaSoft Analysis Pro version 1.0.596 (Bio-Rad).<br>Genotype data: ddPCR or BioMARK HD (Fluidigm)
Data analysis	Flow cytometry data: FACSDiva (version 8.0.2) was analyzed by FlowJo (version 10.10.0)<br>Sequencing reads were mapped to GRCh37 using the Burrows-Wheeler Aligner version: 0.7.17.<br>Error-corrected sequencing: reads with the same unique molecular identifier (UMI) are grouped Pickard (version 2.20.2) and consensus reads were generated using fgbio (version 0.8.1) . Consensus reads were subjected to indel realignment and base quality score recalibration using GATK3 (version 3.8) and recalculation of MD/NM tags using SAMtools (version 1.9). Mutation calling was performed using EBCall (https://github.com/friend1ws/EBCall). Mutations were annotated using ANNOVAR (version 8 June 2020). After mutation calling, read-based variant allele frequencies were calculated based on the reads using the GenomonMutationFilter version 0.2.8.<br>Common for whole-exome and whole-genome sequencing: PCR duplicates were marked using Biobambam version 2.0.87. Errors associated with enzymatic fragmentation were removed using FADE version 0.2.255. Mutation calling was performed using GenomonFisher (version 0.4.4) (https://github.com/Genomon-Project/GenomonFisher). Called mutations were annotated using ANNOVAR (version 8 June 2020). After mutation calling, read-based variant allele frequencies were calculated based on the reads using the GenomonMutationFilter version 0.2.8.<br>Specific to whole-exome sequencing data: Copy number analysis was performed using CNACS (https://github.com/OgawaLabTumPath/CNACS).<br>Spedific to whole-genome sequencing data: Copy number analysis was performed using Control-FREEC version 11.632 and ASCAT_R package version 3.1.157. Chromosome Y loss in male donors were evaluated by calculating the sequence depth of chromosomes X and Y using

SAMtools (version 1.9). Mutational signature analysis was performed using MutationalPatterns version 3.7.0 together with following R packages: ggplot2 (version 3.3.6), biomaRt (version 2.53.3), ccfindR (version 1.17.0), gridExtra (version 2.3), BSgenome.Hsapiens.UCSC.hg19 (version 1.4.3), TxDb.Hsapiens.UCSC.hg19.knownGene (version 3.2.2), BSgenome (version 1.65.2), rtracklayer (version 1.57.0), and NMF (version 0.24.0).
Phylogenetic analysis: Phylogenetic analysis was performed using Sifit (https://github.com/KChen-lab/SiFit). The length of branches were corrected using the "get_corrected_tree" R function downloaded from https://github.com/emily-mitchell/normal_haematopoiesis/tree/main31. Mutations were allocated to branches using the R package "treemut" version 1.1.
ddPCR data: ddPCR data were analyzed using QuantaSoft v1.5.38.1118 software (Bio-Rad). Mutant cell fractions (MCFs) and their credible intervals were generated through Bayesian inference using CmdStan version 2.34.1 based on the number of droplets assigned to the 2D ddPCR quadrants and the number of sorted cells. The Markov Chain Monte Carlo (MCMC) methods implemented in CmdStanR version 0.7.1 were used for parameter estimation.
Statistical analyses were performed using R version 4.2.2. The following open source R packages were used in the analyses presented throughout this paper: magrittr (version 2.0.3), flowCore (version 2.10.0), overlapping (version 2.2), rstan (version 2.32.6), broom (version 1.0.8), bayesplot (version 1.11.1), posterior (version 1.6.1), tidyverse (version 2.0.0), dplyr (version 1.1.4), purr (version 1.0.2), tibble (version 3.2.1), pheatmap (version 1.0.12), stringi (version 1.8.4), stringr (version 1.5.1), ggrepel (version 0.9.5), ggplot2 (version 3.5.1), ggalluvial (version 0.12.5), exactRankTests (version 0.8-35), formattable (version 0.2.1), readxl (version 1.4.3), beeswarm (version 0.4.0), RColorBrewer (version 1.1-3), devtools (version 2.4.5), lmerTest (version 3.1-3), lme4 (version 1.1-35.3), glmmTMB (version 1.1.11), ape (version 5.8), ggtree (version 3.6.2), gplots (version 3.2.0), and spdep (version 1.3-3).

For manuscripts utilizing custom algorithms or software that are central to the research but not yet described in published literature, software must be made available to editors and reviewers. We strongly encourage code deposition in a community repository (e.g. GitHub). See the Nature Portfolio guidelines for submitting code & software for further information.

Data

Policy information about availability of data

All manuscripts must include a data availability statement. This statement should provide the following information, where applicable:

- Accession codes, unique identifiers, or web links for publicly available datasets
- A description of any restrictions on data availability
- For clinical datasets or third party data, please ensure that the statement adheres to our policy

All the DNA sequencing data has been deposited in the Swedish National Data Service (SND) (https://researchdata.se/en, DOI: 10.48723/313d-dd68). Detected mutation list for error-corrected targeted capture sequencing, whole-exome sequencing and single colony whole-genome sequencing, ddPCR result, and codes for data analysis and figure generation are available through SciLifeLab Data Repository (DOI: 10.17044/scilifelab.24745464).
Sequencing data were mapped to combined reference of human genome reference GRCh37 (ftp://ftp.ncbi.nih.gov/genomes/archive/old_genbank/Eukaryotes/vertebrates_mammals/Homo_sapiens/GRCh37/special_requests/GRCh37-lite.fa.gz), Human herpesvirus 4 complete wild type genome (http://www.ncbi.nlm.nih.gov/nuccore/82503188?report=fasta), and the decoy sequence (ftp://ftp.1000genomes.ebi.ac.uk/vol1/ftp/technical/reference/phase2_reference_assembly_sequence/hs37d5cs.fa.gz).
The mutational signature analysis was performed using the combined reference of COSMIC mutational Signatures v2 (https://cancer.sanger.ac.uk/signatures/signatures_v2/) and SBS-blood (https://doi.org:10.1038/s41586-022-05072-7).
736 genes registered in the COSMIC Cancer Gene Census (https://cancer.sanger.ac.uk/census accessed on 2023/03/06) and 95 previously defined myeloid genes (https://doi.org:10.1038/s41586-022-04786-y) were used for the definition of driver mutations.

Research involving human participants, their data, or biological material

Policy information about studies with human participants or human data. See also policy information about sex, gender (identity/presentation), and sexual orientation and race, ethnicity and racism.

Reporting on sex and gender	Information of donor sex is reported in the source data.
Reporting on race, ethnicity, or other socially relevant groupings	All the donors were recruited from Sweden and samples were collected at Karolinska Institutet in Sweden. Donor ethnicity is not available for this study.
Population characteristics	Information of donor age is reported in the source data.
Recruitment	Recruitment of donors was performed under the Stockholm regional ethical review board (EPN 2018/901-31).
Ethics oversight	This study was conducted under the Stockholm regional ethical review board (EPN 2018/901-31).

Note that full information on the approval of the study protocol must also be provided in the manuscript.

Field-specific reporting

Please select the one below that is the best fit for your research. If you are not sure, read the appropriate sections before making your selection.

☒ Life sciences ☐ Behavioural & social sciences ☐ Ecological, evolutionary & environmental sciences

For a reference copy of the document with all sections, see nature.com/documents/nr-reporting-summary-flat.pdf

Life sciences study design

All studies must disclose on these points even when the disclosure is negative.

Sample size	No statistical methods were used to determine sample size since this is an exploratory study. We enrolled all the 93 donors recruited from September 2018 to October 2019.
Data exclusions	All the enrolled donors were included in this study.<br>We excluded whole-genome sequencing data from 21 colonies which showed low depth or skewed peaks of variant allele frequencies in detected mutations.<br>Colonies derived from the same BM sample from HD03 were sequenced on two separate occasions (several years apart) and mutations shared among colonies sequenced on the later occasion were observed which were absent in the earlier sequencing run. These mutations were therefore presumed to be errors induced through long storage and were consequently excluded. Because these errors could lead to inaccurate age estimation, HD03 was also omitted from the statistical analysis for age dating of HSC clones.
Replication	Human CD34+ or CD34+CD19– cells were transplanted into 4 NOD.Cg-PrkdcscidIl2rgtm1Wjl/SzJ (NSG) mice per donor as biological replicates.<br>Mutant cell fractions in B cells after transplantation were confirmed by performing ddPCR using biological replicates for sorting.<br>We validated all the mutations detected by error corrected targeted capture sequencing with >2% VAF driver mutations and >1% non-driver mutations by ddPCR.
Randomization	Randomization is not relevant to this study since all the donors were healthy and subjected to screening of mutations.
Blinding	Blinding is not relevant to this study.

Reporting for specific materials, systems and methods

We require information from authors about some types of materials, experimental systems and methods used in many studies. Here, indicate whether each material, system or method listed is relevant to your study. If you are not sure if a list item applies to your research, read the appropriate section before selecting a response.

Materials & experimental systems

n/a	Involved in the study	
☐	☒	Antibodies
☐	☒	Eukaryotic cell lines
☒	☐	Palaeontology and archaeology
☐	☒	Animals and other organisms
☐	☒	Clinical data
☒	☐	Dual use research of concern
☒	☐	Plants

Methods

n/a	Involved in the study	
☒	☐	ChIP-seq
☐	☒	Flow cytometry
☒	☐	MRI-based neuroimaging

Antibodies

Antibodies used	Information on the antibodies used in this study, including dilutions, catalog numbers, and lot numbers, is provided in Supplementary Table 5.
Validation	All antibodies used in the study were obtained from commercial vendors and were validated by their manufacturers for the application (flow cytometry) and species (mouse) used in this study. Furthermore, all antibodies used have been individually titrated prior to use to identify their optimal concentration in the required application. The specificity of staining was controlled based on simultaneous analysis of cell populations known to lack expression of the relevant antigens. All experiments included fluorescence-minus-one (FMO) controls and, where possible, staining panels included internal controls (known negative and positive populations) to validate specific antibody signals.

Eukaryotic cell lines

Policy information about cell lines and Sex and Gender in Research

Cell line source(s)	Jurkat and K562: both from ATCC
Authentication	Authenticated cell lines were purchased from ATCC.
Mycoplasma contamination	Cells were regularly tested for mycoplasma contamination and were confirmed negative before experiments.
Commonly misidentified lines<br>(See ICLAC register)	Not used in this study.

Animals and other research organisms

Policy information about studies involving animals; ARRIVE guidelines recommended for reporting animal research, and Sex and Gender in Research

Laboratory animals	NOD.Cg-PrkdcscidIl2rgtm1Wjl/SzJ (NSG) mice from the Jackson Laboratory
Wild animals	No wild animals were used in this study.
Reporting on sex	Female mice were used as described in the methods.
Field-collected samples	Not relevant for this study.
Ethics oversight	All mouse experiments were performed at Karolinska Institutet in Sweden according to the guidelines and obtained permissions from the ethics committees at Stockholms Djurförsöksetiska Nämnd (17978-18 with amendments 18539-21).<br>Mice were maintained in individually ventilated cages with a 12/12 h light/dark cycle, at 22 ± 1 °C and 50% relative humidity.

Note that full information on the approval of the study protocol must also be provided in the manuscript.

Clinical data

Policy information about clinical studies

All manuscripts should comply with the ICMJE guidelines for publication of clinical research and a completed CONSORT checklist must be included with all submissions.

Clinical trial registration	Not a clinical trial, and not relevant for the others
Study protocol	*Note where the full trial protocol can be accessed OR if not available, explain why.*
Data collection	*Describe the settings and locales of data collection, noting the time periods of recruitment and data collection.*
Outcomes	*Describe how you pre-defined primary and secondary outcome measures and how you assessed these measures.*

Plants

Seed stocks	*Report on the source of all seed stocks or other plant material used. If applicable, state the seed stock centre and catalogue number. If plant specimens were collected from the field, describe the collection location, date and sampling procedures.*
Novel plant genotypes	*Describe the methods by which all novel plant genotypes were produced. This includes those generated by transgenic approaches, gene editing, chemical/radiation-based mutagenesis and hybridization. For transgenic lines, describe the transformation method, the number of independent lines analyzed and the generation upon which experiments were performed. For gene-edited lines, describe the editor used, the endogenous sequence targeted for editing, the targeting guide RNA sequence (if applicable) and how the editor was applied.*
Authentication	*Describe any authentication procedures for each seed stock used or novel genotype generated. Describe any experiments used to assess the effect of a mutation and, where applicable, how potential secondary effects (e.g. second site T-DNA insertions, mosiacism, off-target gene editing) were examined.*

Flow Cytometry

Plots

Confirm that:

☒ The axis labels state the marker and fluorochrome used (e.g. CD4-FITC).

☒ The axis scales are clearly visible. Include numbers along axes only for bottom left plot of group (a 'group' is an analysis of identical markers).

☒ All plots are contour plots with outliers or pseudocolor plots.

☒ A numerical value for number of cells or percentage (with statistics) is provided.

Methodology

Sample preparation	Viably frozen bone marrow mononuclear cells were thawed in a 37°C water bath and washed with Dulbecco ́s phosphate buffered saline (PBS, Gibco) supplemented with 20% FCS (Sigma-Aldrich) and 100 µg/ml DNase I, Bovine Pancreas (Sigma-Aldrich). For human cells transplanted into NSG mice, BM was isolated 10-14 weeks after transplantation.<br>After 5 minutes incubating in FcR Blocking Reagent, human (Miltenyi Biotec), cells were stained with the fluorescently conjugated monoclonal antibodies for 15 minutes at 4 °C.<br>Stained cells were washed with PBS supplemented with 5% FCS and 2mM EDTA (Invitrogen) and was added with DAPI

(Invitrogen) for HSPC panel or 7AAD for mature cell panel (Sigma-Aldrich) for identifying live cells just before FACS analysis.

Instrument	All flow cytometry experiments were performed on a FACSAria Fusion (BD Biosciences).
Software	FACSDiva (version 8.0.2), FlowJo (version 10.10.0), and R package flowCore (version 2.10.0).
Cell population abundance	For all the cell sorting, the purities of targeted cell populations were checked prior to cell sorting was done. For populations with ≥50 cells were sorted for purity evaluation, we achieved >97% purity in live cell gating (n=83).<br>Single cell sorting was performed with index sorting which allows prospective analysis of the cell surface expression of the single cell sorted. Accuracy of single cell deposition in 96 well plates was validated using 488 nm fluorescent beads.
Gating strategy	FSC-A/SSC-A was used for gating mononuclear cells. Doublets were excluded. 7AAD or DAP positive cells were gated out to exclude non-viable cells.<br>Live cells were gated according to the following expression markers.<br>Haematopoietic stem cells (HSCs): Lineage–CD34+CD38–CD90+CD45RA–<br>Megakaryocyte progenitor cells (MkP): Lineage–CD34+CD38+CD41a+<br>Erythroid progenitor cells (EP): Lineage–CD34+CD38+CD41a–CD123–CD45RA–<br>ProB cells (ProB): Lineage–CD34+CD19+<br>Myeloid cells: CD14+CD33+CD3–CD19–CD56–<br>B cells: CD19+CD3–CD33–CD56–<br>T cells: CD3+CD8a+CD4–CD19–CD33–CD56– and CD3+CD4+CD8a–CD19–CD33–CD56–<br>Lineage markers include CD2, CD3, CD4, CD7, CD8a, CD10, CD11b, CD14, CD19, CD20, CD56, and CD235a.<br>Isolation of humane cells after transplantation into NSG mice: Human myeloid cells (mTer119–mCD41–mCD45–hCD235ab–hCD45+CD33+CD19–CD36–) and human B cells (mTer119–mCD41–mCD45–hCD235ab–hCD45+CD33–CD19+CD36–)

☒ Tick this box to confirm that a figure exemplifying the gating strategy is provided in the Supplementary Information.
